# Photosystem I and ZIF‑8 Interfacing: Entrapment and Immobilization

**DOI:** 10.1021/acs.inorgchem.4c05441

**Published:** 2025-05-20

**Authors:** Kathrin L. Kollmannsberger, Sarah V. Dummert, Erling Thyrhaug, Pritam Banerjee, Feng Liu, Dario Leister, Joerg Jinschek, Jürgen Hauer, Roland A. Fischer, Julien Warnan

**Affiliations:** † Chair of Inorganic and Metal-Organic Chemistry, Department of Chemistry, TUM School of Natural Sciences, 9184 Technical University of Munich, Lichtenbergstr. 4, 85748 Garching, Germany; ‡ Professorship of Dynamic Spectroscopy, Department of Chemistry and Catalysis Research Center (CRC), TUM School of Natural Sciences, Technical University of Munich, Lichtenbergstr. 4, 85748 Garching, Germany; § National Centre for Nano Fabrication and Characterization (DTU Nanolab), 5205 Technical University of Denmark, Fysikvej 307, DK-2800 Kongens Lyngby, Denmark; ∥ Faculty of Biology, 9183 Ludwig-Maximilians-Universität München, Großhaderner Str. 2-4, 82152 Planegg-Martinsried, Germany

## Abstract

In this study, we explore the interfacing of Photosystem I (PSI) with the metal–organic framework (MOF) ZIF-8 (ZIF = zeolitic imidazolate framework) through encapsulation and surface immobilization methods, aimed at stabilizing PSI through biohybrid composite formation. PSI was successfully encapsulated within ZIF-8 (PSI@ZIF-8) and immobilized on ZIF-8 surfaces (PSI/ZIF-8) using a one-pot synthesis and surface impregnation technique, respectively. Characterization techniques including powder X-ray diffraction, Fourier transform infrared spectroscopy, and high-angle annular dark-field scanning transmission electron microscopy confirmed the formation and first-of-its-kind nanoscale visualization of the PSI/ZIF-8 composites. Spectroscopic analysis revealed that while PSI encapsulation resulted in minor structural changes potentially from scaffolding-induced stress and MOF building blocks, the overall protein integrity was maintained. Our study demonstrates that, in contrast to surface interfacing, ZIF-8 encapsulation provides a protective environment for PSI, enhancing its stability and retaining its functional properties, thereby offering an auspicious approach for the development of biohybrid materials in semi-artificial photosynthesis and other biotechnological applications.

## Graphical abstract

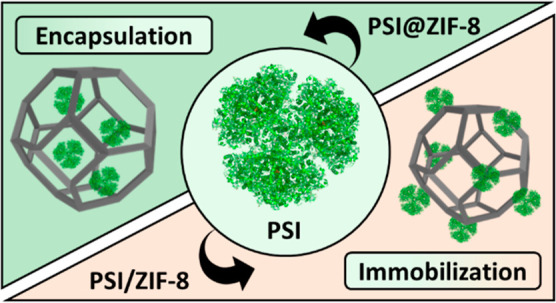

## Introduction

Synthetic biology has greatly expanded the ability to modify natural systems for enhanced functionality and the creation of artificial biological systems.^1^ However, the complexity of its machinery and metabolic pathways imposes limitations on engineering flexibility. Biohybrid materials connect the advancing fields of synthetic biology and material science, providing a platform for the development of biocatalytic systems and tailoring the construction of biologically inaccessible pathways.^2–5^

Photosystem I (PSI) is an archetypal example of a biohybrid material-relevant enzyme. As a chlorophyll-rich transmembrane multi-protein complex, it plays a crucial role in photosynthesis by driving light-activated charge separation and electron transport in plants and cyanobacteria. It is a large (∼1000 kDa), disc-like membrane complex with desirable properties, such as broad visible light absorption, nearly quantitative quantum efficiency under low light intensity, a charge separation potential of ∼1 V, and an energy efficiency of approximately 58%. However, leveraging these properties outside of PSI’s natural environment presents enormous challenges, due to its susceptibility to aggregation, high sensitivity to environmental factors, such as temperature and radiation, and stability as well as activity loss upon isolation.^6–9^

Various stabilization strategies, including cross-linking, enzyme functionalization, medium engineering, and immobilization, have been explored to address these challenges.^10–13^ Immobilization, in particular, is advantageous for practical applications due to the stabilization, reusability and harnessing of the (electro)­catalytic activity it grants.^13,14^ However, the weak optical absorption presented by a single PSI monolayer (<1% of incident light at 680 nm) represents a common challenge in these studies.^15^ To overcome this, researchers have deposited multi-layer assemblies of PSI on various light absorbing surfaces, including p-doped silicon and graphene.^16–21^ Additionally, the use of mesoporous electrodes, hydrogels, conductive polymers, and combinations of DNA binders and complementary enzyme assemblies has been investigated.^22–27^ Alternatively, recent works pursue designing tailored structural microenvironments that mimic the native thylakoid membrane, such as incorporating PSI into lipid bilayers, to study and ultimately control how these environments affect energy transfer within the chlorophyll network.^28^

Here we investigate associating PSI with a metal–organic framework (MOF). MOFs are highly organized crystalline structures made from inorganic nodes connected by organic linkers, featuring tunable network architectures, high permeability and large surface areas. Specifically, the zeolitic imidazolate framework-8 (ZIF-8) is composed of zinc nodes and 2-methylimidazole linkers, offering cavities and apertures size-amenable to the guests, while pristine, defect-free ZIF-8 features a pore size of 11.6 Å and an aperture of 3.4 Å.^29^ ZIF-8 is transparent to visible light, highly porous, chemically and thermally stable, and can easily be synthesized under biocompatible conditions (e.g., in aqueous solution at room temperature) within minutes, enabling a one-step, in situ process for guest encapsulation.^30,31^ Moreover, ZIF-8 demonstrates good biocompatibility and has previously been shown to act as a microenvironmental protective shield, helping to prevent the denaturation of encapsulated enzymes.^32–35^ Past examples of enzyme@ZIF-8 host–guest systems revealed that hydrophobic interactions and the coordination of histidine and cysteine residues facilitate the protein scaffolding process.^36–39^ Controlling the environment of encapsulated proteins, ZIF-8 can enhance their stability and activity, thereby protecting them from denaturation under extreme conditions. Nonetheless, the effects of MOF encapsulation on large membrane proteins are not yet fully understood, requiring further investigations.^40^

While previous studies have reported layer-by-layer assembly of PSI within MOFs, herein the support-effect of ZIF-8 on the enzyme PSI is investigated through monitoring the entrapment vs. surface immobilizing of PSI.^22,41,42^ A fast, in situ, and straightforward synthetic encapsulation approach, denoted as one-pot scaffolding yielding PSI@ZIF-8, is presented and compared to a facile PSI/ZIF-8 surface impregnation approach, named PSI/ZIF-8 (Scheme 1). The impact of three-dimensional confinement and the surface support of ZIF-8 on PSI properties was investigated, including chemical stability, fluorescence activity and UV/Vis absorption. High-angle annular dark-field (HAADF) scanning transmission electron microscopy (STEM) imaging unlocked visualizing the PSI/ZIF-8 hybrid materials to assess the location and distribution of the enzyme. Spectroscopic investigations revealed similar behavior upon interfacing but divergences in the release and protectivebehavior of the two approaches.

**Scheme 1:**
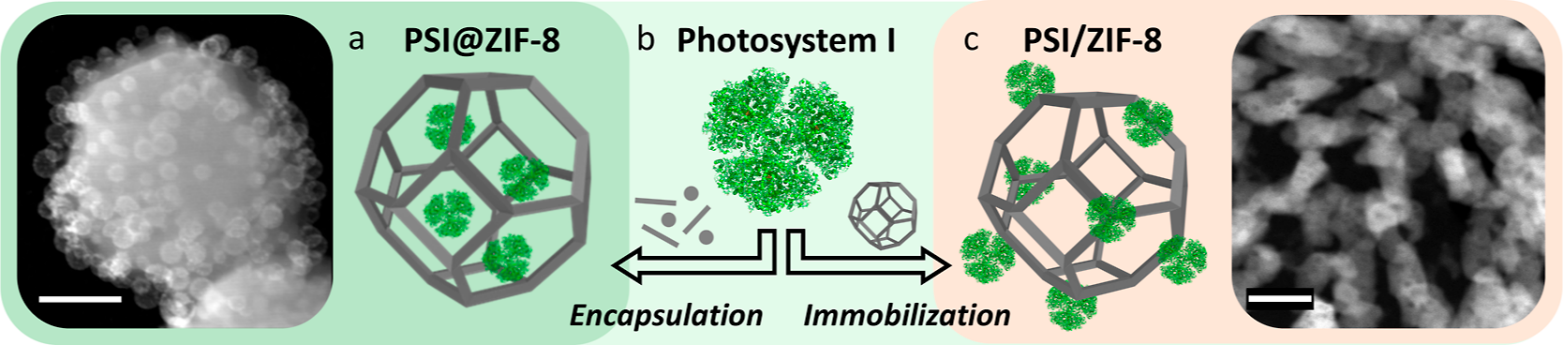
PSI/ZIF-8 hybridization via an encapsulation or immobilization strategy yielding PSI@ZIF-8 (a) or PSI/ZIF-8 (c), respectively.^a^

a For encapsulation, Photosystem I (PSI, b) is combined with the ZIF-8 building blocks in a so-called one-pot scaffolding. The immobilization is achieved by combining preformed ZIF-8 with PSI, aiming for surface-anchoring. PSI@ZIF-8 and PSI/ZIF-8 are displayed schematically as well as through representative HAADF-STEM images (scale bars: 50 nm).

## Experimental Section

### General Information

All chemicals were purchased from commercial suppliers and used without purification. PSI is stored at −80 °C in 100 μL vials in the resuspension buffer (30 mM Tricine-NaOH pH 8, 15 mM NaCl, small remnant of sucrose, detergent β-DDM). For the work with PSI-containing compounds, light exposure was minimized. If not stated otherwise PSI, PSI@ZIF-8 or PSI/ZIF-8 are dissolved or suspended in 50 mM phosphate buffer (pH 7.4) for UV/Vis spectroscopy.

### Synthetic Procedures

#### Growth and Purification of Photosystem I

The PSI purification protocol was adapted from Dobson et al.^43^ Cells from the model cyanobacterium Synechocystis sp. PCC6803 (wild type) were grown in BG11 liquid medium, supplemented with 5 mM glucose under continuous white light (50 μmol photons m^–2^ s^–1^) at 30 °C. Cells in the log phase were harvested by centrifugation at 5000*g* for 10 min at room temperature, and the cell pellets were stored at −80 °C for further use. For protein purification, frozen cell pellets were thawed and resuspended in cold STN1 buffer (30 mM Tricine-NaOH pH 8, 15 mM NaCl, 0.4 M sucrose) and glass beads (212–300 μm, 425–600 μm, Sigma) were added. Cells were broken using a Tissue Lyser II bead mill (Qiagen) for 5 cycles, each cycle consisting of 3 min at 30 Hz, followed by 5 min cooling on ice. The lysate was cleared of cell debris and glass beads by centrifuging at 5000*g* for 5 min at 4 °C. Thylakoid membranes were pelleted by ultracentrifugation using a Beckman SW40i rotor at 40,000 rpm for 1 h at 4 °C. The membranes were then resuspended and incubated in STN2 buffer (30 mM Tricine-NaOH pH 8, 150 mM NaCl, 0.4 M sucrose) on ice for 30 min. The ultracentrifugation was repeated to pellet thylakoid membranes again. The membranes were resuspended in resuspension buffer (30 mM Tricine-NaOH pH 8, 15 mM NaCl) and *n*-dodecyl β-maltoside (DDM, ANAGRADE) was added to achieve a mass ratio of 15:1 DDM-to-chlorophyll. The samples were gently mixed by pipetting and incubated on ice for 30 min. Any insoluble materials were removed by the same ultracentrifugation, and the supernatant was collected and applied to an ion-exchange column (Toyopearl DEAE-650M, 5 mL, TOSOH BIOSCIENCE) on Äkta. Proteins were eluted with a linear NaCl gradient from 15 mM to 350 mM in a buffer of 30 mM, Tricine-NaOH (pH 8) and 0.2% DDM. The dark green fractions were collected and loaded on 10–30% sucrose gradient (30 mM Tricine-NaOH pH 8, 15 mM NaCl), followed by centrifugation at 36,000 rpm for 16 h at 4 °C using a Beckman SW40i rotor. The lower green bands, corresponding to the PSI trimer, were collected and 8% PEG3350 was added to precipitate the protein. Precipitated protein was resuspended in resuspension buffer and stored at −80 °C for further use.

#### ZIF-8

First, 67.0 mg zinc acetate dihydrate (0.305 mmol, 1.0 equiv) were dissolved in 20 mL Milli-Q water. Subsequently, this solution was combined with an aqueous solution of 2.47 g 2-methylimidazole (30.1 mmol, 98 equiv) in 20 mL Milli-Q water. The mixture was placed in the dark for 3 h at room temperature. The white precipitate was separated by centrifugation at 5000*g* for 10 min. The obtained product was washed with Milli-Q water (3 × 10 mL). Drying under dynamic vacuum yielded the white powder.

#### PSI@ZIF-8

First, 12.3 mg zinc acetate dihydrate (0.056 mmol, 1 equiv) and 220.8 mg 2-methylimidazole (2.69 mmol, 48 equiv) were dissolved in 200 μL and 1800 μL Milli-Q water, respectively. Then, the zinc solution was incubated with 10–100 μL PSI (2.44 μg μL^–1^) for 20 min, before adding the 2-methylimidazole solution and stirring for 10 min. The samples were aged for 1 h and then washed with (Milli-Q water, 3 × 2 mL), yielding the product as a green, wet solid. Depending on the sample, a washing procedure with SDS (500 μL, 4 wt %) was conducted subsequently.

#### PSI/ZIF-8

First, 12.0 mg ZIF-8 (0.05 mmol) were suspended in 1800 μL Milli-Q water via ultrasonication. 24 μL–48 μL PSI (0.94 μg μL^–1^) was added to the suspension and the mixture was stirred in the dark for 10 min. After centrifugation at 5000*g*, the product was obtained as a green, wet solid. Depending on the sample, a washing procedure with SDS (500 μL, 4 wt %) was conducted subsequently.

### Characterization

#### UV/Vis Stability Tests

10 μL of PSI (0.94 μg μL^–1^) were incubated with 500 μL of the respective reaction medium. A UV/Vis spectrum was measured for each sample directly after mixing (0 min) and after 30, 120, 300 min. The tested reaction media include: phosphate buffer (50.0 mM, pH 7.4), 2-MeIm (15.0 mM, high concentration: 1.49 M), Zn­(OAc)_2_ (0.27 M), acetate buffer (500 mM; pH 5.3) and SDS (4 wt %).

#### Powder X-ray Diffraction

PXRD measurements were performed on a silicon single-crystal wafer using *Bragg Brentano* geometry in a *Rigaku* MiniFlex 600 C diffractometer. X-ray Cu Kα radiation (λ_1_—1.5406 Å, λ_2_—1.5444 Å, *I*_2_/*I*_1_—0.5) was used, and K_β_ radiation was removed by a Ni-filter. The measurement range, unless stated otherwise, was from 2.0° to 50.0/90.0° (2θ) with a step size of 0.010° and a scan rate of 5° per minute.

#### IR Spectroscopy

Attenuated total reflection Fourier transform infrared (ATR-FTIR) spectra were collected on a *PerkinElmer Frontier* FTIR spectrometer with a diamond ATR crystal.

#### Zeta Potential

A 5 μL PSI sample was diluted with 950 μL Milli-Q, transferred into the disposable folded capillary cell (DTS1070) of a *Malvern* Zetasizer Nano ZS and immediately measured. The value was averaged over three independent values. For the measurement in the presence of Zn^2+^, 5 μL of PSI (2.44 μg μL^–1^) were added to a 15 mM Zn­(OAc)_2_ solution before measurement.

#### HAADF-STEM Imaging

The high angle annular dark field (HAADF) scanning transmission electron microscope (STEM) image was acquired using a *ThermoFisher* TITAN transmission electron microscope (TEM), equipped with field emission gun (FEG), and operated at 300 keV. A beam convergence angle of 10 mrad, a camera length of 245 mm with collection angle of 63–200 mrad, and a beam current of 60 pA was used. The HAADF-STEM images have a size of 1024 × 1024 pixels and were acquired with a dwell time of 12 μsec per pixel.

#### UV/Vis Spectroscopy

The UV/Vis spectra were measured using alternatively an *Agilent Technologies* Cary 60 or a *PerkinElmer* lambda 365 UV/Vis spectrometer. All spectra were measured at room temperature in 10 mm pathlength SUPRASIL quartz glass cuvettes. The background absorption of solvents was subtracted from the spectra.

#### Diffuse-Reflectance UV/Vis Spectroscopy

The diffuse-reflectance UV/Vis spectra were measured on a *Shimadzu* UV-3600 Plus with integrating sphere unit. The samples were put between two quartz microscope slides and their reflection was measured from 200 to 800 nm. Barium sulfate was used as a reference.

#### Fluorescence Spectroscopy

Solutions and dispersions of PSI, PSI/MOF and PSI@MOF suitable for fluorescence experiments were freshly prepared and immediately transferred to a 1 cm pathlength quartz cuvette before each measurement. To avoid artifacts due to self-absorption, the PSI absorption was kept below 0.05 at the absorption maximum near 670 nm. Dispersions of MOF-embedded PSI were stirred using a magnetic stir-bar to avoid precipitation, and the temperature was kept at 20 °C. The UV/Vis absorption spectra of all samples were recorded immediately prior to fluorescence experiments.

The UV/Vis absorption spectra of all samples were recorded prior to fluorescence experiments. In-solution absorption spectra were recorded in the sample cell on a *PerkinElmer* lambda 365 UV/Vis spectrometer, using the relevant buffer system as a background.

Fluorescence spectra were measured on an *Edinburgh Instruments* FS5 spectrofluorometer equipped with computer-controlled calcite polarizer prisms and dual-grating monochromators. The sample chamber was equipped with an electronic Peltier element and a magnetic stirrer. The signal was detected at right angle to the excitation, while the transmission through the sample was monitored at regular intervals to exclude sample degradation. The detected signal was automatically corrected for detector sensitivity and excitation intensity.

## Results and Discussion

### Synthesis and Characterization of PSI@ZIF-8

PSI@ZIF-8 samples were produced by first incubating an aliquot of PSI (from Synechocystis sp. PCC6803) in a Zn­(OAc)_2_ solution for 20 min.^44^ A 2-methylimidazole (2-MeIm) solution was added under stirring and the mixture was reacted for 10 min. The powders were left 1 h in the dark without stirring, as an aging procedure, before washing with Milli-Q water and concentration of PSI@ZIF-8 as a green slurry via centrifugation.

Powder X-ray diffraction (PXRD), thermogravimetric analysis (TGA), Fourier transform infrared (FTIR), and diffuse reflectance ultraviolet–visible spectroscopy (DR-UV/Vis) analysis were conducted on the dried PSI@ZIF-8 composite powders. The PXRD pattern of the powder is similar to that of simulated ZIF-8 structures, confirming the formation of crystalline ZIF-8 (Figure 1a). The FTIR spectrum of PSI@ZIF-8 resembles that of the pristine ZIF-8 without additional PSI-related features (Figure S1a), indicating modest enzyme loading.^41^ The TGA of pristine ZIF-8 and PSI@ZIF-8 revealed overall good thermal stability (Figure S1b). For pristine ZIF-8, a weight-loss of 20% below 250 °C is associated with residual solvent and 2-MeIm evaporation, and is followed by decomposition, which is in accordance with literature.^45^ To account for this initial loss of volatile components and better assess the mass loss attributable to PSI, the curves were normalized to the residual weights at 260 °C. Pristine ZIF-8 displays a smooth, single-step decomposition process between 360 and 630 °C, consistent with the thermal breakdown of the ZIF-8 framework. In contrast, PSI@ZIF-8 exhibits a broader and more complex decomposition profile, spanning 260–715 °C and featuring multiple degradation steps. This behavior is characteristic of hybrid materials containing biological macromolecules and may reflect the intricate breakdown of the encapsulated enzyme and scaffolding framework, as well as possible interactions between the two. Notably, the onset of framework decomposition appears earlier for PSI@ZIF-8 than for pristine ZIF-8, and the decomposition extends over a wider temperature range. This may indicate a reduced thermal stability of the ZIF-8 matrix in the composite, possibly related to PSI-induced structure defects or bulk-inherent variations in PSI content and crystal morphology. A comparison of the normalized residual masses at 800 °C reveals a greater weight loss for PSI@ZIF-8 (32.3% residual mass) than for pristine ZIF-8 (35.9% residual mass), consistent with a higher proportion of decomposable organic matter relative to thermally stable inorganic Zn residues.

**Figure 1:**
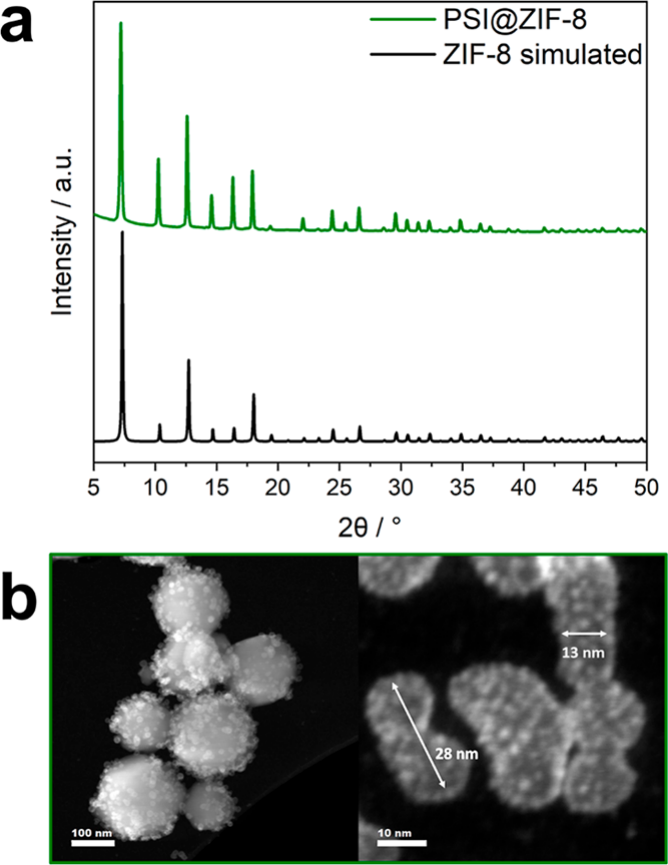
(a) PXRD of PSI@ZIF-8 and a simulated ZIF-8 pattern as reference, (b) HAADF-STEM images of as-synthesized PSI@ZIF-8 with encapsulated PSI units (note that PSI units also feature on the outer surface of ZIF-8 crystals (left)). The dimensions of individual enzyme units are indicated by arrows (right).

Electron microscopy has rarely been employed to analyze enzyme-MOF materials, as direct imaging of proteins of MOF-encapsulated proteins is difficult due to the low contrast between the organic material and organic–inorganic matrix.^40,46–48^ HAADF-STEM imaging was employed to further investigate the PSI@ZIF-8 composite. Broad particle screenings revealed PSI units adhering to the surfaces of ZIF-8 crystals (Figures 1b and S2), while smaller crystals with PSI clearly encapsulated within the ZIF-8 framework were also observed (Figure S3). The high image resolution enabled us to directly observe and extract the size of the enzymes and even differentiate between the PSI top and side view (Figure 1b). The diameter of the PSI, based on its crystal structure, has been reported as 22 nm with a height of 10 nm.^22,49^ In the images presented here, the large (∼28 nm) and smaller (∼13 nm) spherical species can be assigned to the top- and side-viewed PSI, respectively. The small size differences relative to the theoretical dimensions may originate from a less constrained environment or protein swelling. Although electron microscopy is an important tool to analyze the oligomerization state of PSI or to visualize the stabilizing agents, this is, to the best of our knowledge, the first reported electron microscopy imaging of MOF-supported PSI.^24,50,51^

While these analyses support the successful attachment of PSI on the MOF, it remains unclear how much of the enzyme is located inside the ZIF-8 crystallites. By averaging the number and dimensions of PSI units in various HAADF-STEM images (Figures 2a and S2), it can be estimated that 45–50 enzyme units are present on the surface of a 50 nm ZIF-8 crystal, with an average diameter of 23.1 ± 4.1 nm and an average height of 13.0 ± 1.6 nm. To remove this residual surface-bound PSI, PSI@ZIF-8 was immersed in 4 wt% aqueous sodium dodecyl sulfate (SDS) for approximately 5 min. The MOF structure remained largely unaffected by this procedure (Figure S4). DR-UV/Vis spectroscopy was conducted pre- and post-washing, revealing a ∼22% decrease in intensity, with no obvious change in the shape of the spectrum (Figure S5). Further, HAADF-STEM analysis of the SDS-washed samples confirmed quantitative removal of surface-bound PSI and revealed the fraction of encapsulated PSI (Figure 2b). The images suggest an average loading of 4 PSI units within a 200 nm ZIF-8 crystal, yielding a surface-loading-to-encapsulation ratio of 50:1 (Figures 2 and S6).

**Figure 2:**
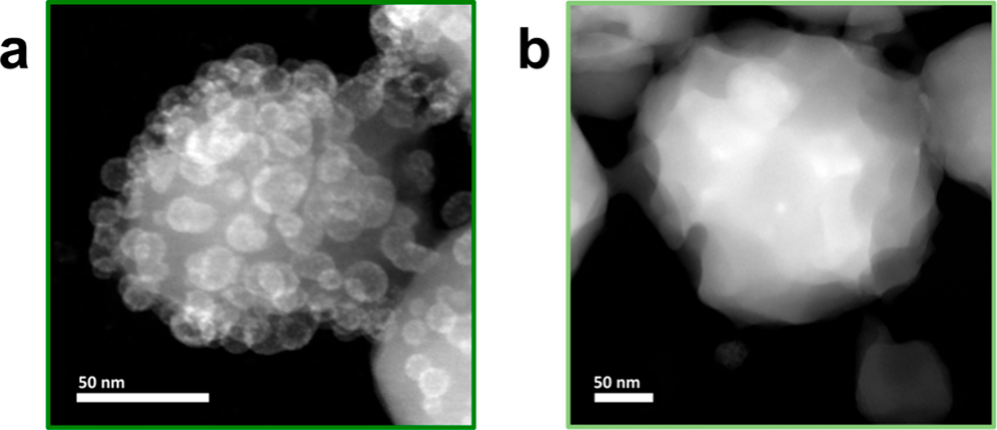
(a) HAADF-STEM images of PSI@ZIF-8 pre- and (b) post-washing with SDS. Note: In the as-synthesized material, multiple PSI units also feature on the outer surface of ZIF-8 crystals. After their removal via SDS washing, encapsulated PSI appears as bright regions within ZIF-8.

To investigate the state of the PSI protein integrity within ZIF-8, DR-UV/Vis measurements were performed and compared to the UV/Vis spectrum of PSI in phosphate buffer (50 mM, pH 7.4) (Figure 3a). Mineralization of PSI to form the encapsulated PSI@ZIF-8, resulted in a hypsochromic shift of the absorption spectrum relative to that of PSI in buffer (B-band: −5 nm, Q-band: −9 nm). Nevertheless, the spectrum clearly retains its overall shape, showing the characteristic chlorophyll *a* (Chl_a_) B- and Q-band absorption features of PSI at ∼433 and ∼670 nm, as well as the carotenoid absorption around 500 nm. Notably, the relative intensity of the B band decreased, lowering the B- to Q-band ratio by ∼20% to roughly 1:1.

To further evaluate the state of PSI within the MOF, we conducted fluorescence spectroscopy experiments on a PSI@ZIF-8 sample dispersed in phosphate buffer (50 mM, pH 7.4), as shown in Figure 3b. In accordance with the UV/Vis absorption spectrum and prior reports for confined PSI materials,^22^ both fluorescence and the Q-band region of the excitation spectra exhibited a visible blue shift (fluorescence: −8 nm, excitation: −10 nm). Although broadly similar, the fluorescence excitation spectrum shows several notable differences compared to the in-solution spectra of pristine PSI. In particular, the relative intensity of the Q-band is increased (∼7%), the vibronic structure of the B-band is altered, and we observed a loss of the carotenoid features around 500 nm. These differences imply that the observed fluorescence is not the weak PSI emission, but rather from a minor population of decomposition products resulting from the MOF encapsulation. In particular, the observed spectra are similar to those of pheophytin *a*, which is readily formed from Chl_a_.^52–54^

**Figure 3:**
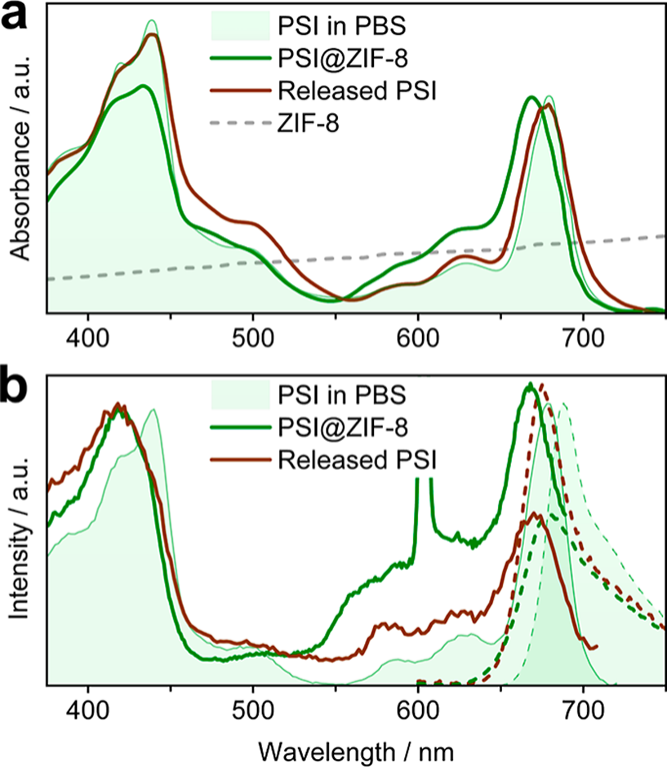
(a) Normalized (DR)-UV/Vis spectra of PSI in phosphate buffer (green shaded area), encapsulated in ZIF-8 (green line) and after release from the ZIF-8 matrix (brown line). The scattering spectrum of ZIF-8 is shown as dashed line. (b) Normalized fluorescence (dashed lines) and fluorescence excitation (full lines) of PSI in phosphate buffer (green shaded area, λ_Ex_ = 425 nm, λ_Em_ = 730 nm), encapsulated in ZIF-8 (green, λ_Ex_ = 440 nm, λ_Em_ = 740 nm) and after release from the ZIF-8 matrix (brown, λ_Ex_ = 440 nm, λ_Em_ = 720 nm). The sharp feature at ∼600 nm in the fluorescence excitation spectrum of PSI@ZIF-8 is caused by Raman scatter from the ZIF-8 matrix.

Next, the PSI enzyme was carefully released from the ZIF-8 matrix and analyzed using UV/Vis absorption, excitation, and fluorescence spectroscopy (Figure 3) to evaluate the degree of denaturation of PSI post-entrapment. For this, PSI@ZIF-8 was exposed to ∼500 μL of acetic acid buffer (500 mM, pH 5.3), which led to MOF degradation within a few minutes, resulting in a clear, slightly green solution. The UV/Vis spectrum of the recovered PSI was immediately recorded and, beyond a slight increase in spectral line widths, closely resembles that of pristine PSI in phosphate buffer (Figure 3a). For both the B- and Q-bands, the absorption maxima returned to the original wavelengths, and their ratio was restored, with only a slight decrease in overall intensity (∼5%). The broadening of the spectral lines suggests that the acidified buffer solution containing ZIF-8 digestion products poses a more complex and strongly interacting environment than the pure phosphate buffer. In contrast, the fluorescence excitation spectra of PSI failed to revert after ZIF-8 digestion (Figure 3b). Instead, the B- to Q-band ratio is strongly increased, due to a ∼40% intensity loss of the Q-band, and the carotenoid features are entirely absent. This gives further credence to the hypothesis that PSI mineralization leads to emission properties dominated by a minor population of decomposition products with spectral similarities to pheophytin.^52–54^ Details of all spectral data (UV/Vis and fluorescence excitation and emission) are documented in Table S1.

Overall, the recovered DR-UV/Vis spectrum suggests that PSI is largely preserved throughout both, encapsulation and release. The mismatch between fluorescence and absorption, however, implies pigment loss accompanied by Mg leaching from a small fraction of the protein population during the MOF scaffolding process. This suggests that considerable care should be taken when analyzing emission spectra from these structures, as the fluorescence from solvated impurities such as pheophytin (quantum yield (QY) ∼14% at room temperature)^55^ can be much higher than that of intact PSI (QY ≤ 1% at RT), where emission is efficiently quenched by charge separation in the reaction center.^56,57^

### Synthesis and Characterization of PSI/ZIF-8

For the PSI interfacing with ZIF-8, pre-synthesized ZIF-8 powder was suspended in Milli-Q water before an aliquot of PSI was added, and the mixture was incubated for 10 min. After centrifugation, PSI/ZIF-8 was obtained as a green solid. The supernatant showed no visible light absorption (Figure S7), indicating quantitative attachment and a strong affinity of PSI for ZIF-8. HAADF-STEM analysis of the PSI/ZIF-8 sample (Figure 4a) revealed immobilized, albeit aggregated PSI on the ZIF-8 crystals. The immobilization of the PSI on ZIF-8 works quantitatively and the enzyme is distributed heterogeneously on the support.

SDS treatment of PSI/ZIF-8 was performed by immersing the samples in 4 wt % SDS for ∼5 min, followed by centrifugation. The detachment of PSI from the ZIF-8 surface was quantified via DR-UV/Vis analysis of pre- and post-washing samples, revealing a removal efficiency of approximately 60% (Figure S8). Further SDS washings could achieve complete PSI removal, underscoring the lower stabilization compared to PSI@ZIF-8. The corresponding PXRD pattern attests that ZIF-8 crystallinity is globally unaffected by the PSI immobilization and subsequent SDS washing procedure (Figure 4b). HAADF-STEM images of SDS-washed PSI/ZIF-8 samples revealed no visible PSI at the MOF surface (Figure S9). Similar to PSI@ZIF-8, DR-UV/Vis analysis of the unwashed composite showed a hypsochromic shift of the B- and Q-band (3 and 10 nm, respectively) along with a slight broadening of signals compared to the PSI in buffer. However, in contrast to the encapsulated species, the intensities remain largely unaffected (Figure 5a). Thus, while the characteristic absorption features of PSI were largely preserved in the PSI@ZIF-8 composite, the UV/Vis spectrum of PSI/ZIF-8 suggests an even higher degree of structural integrity. This is further evidenced by the fluorescence emission and excitation measurements of PSI/ZIF-8 suspended in phosphate buffer (50 mM, pH 7.4). Both displayed high comparability with that of PSI in solution (Figure 5b), with the Q-band region less shifted towards the blue in the emission spectrum (−2 nm, excitation: −1 nm). In the excitation spectrum, the vibronic structure and position of the B-band are largely retained, while the Q-band signal shows no significant changes apart from a small increase of intensity (∼10%), and the carotenoid features are preserved. The strong spectroscopic similarities of PSI/ZIF-8 to pristine PSI suggest that the interfacing with the MOF support induces only minimal decomposition.

**Figure 4:**
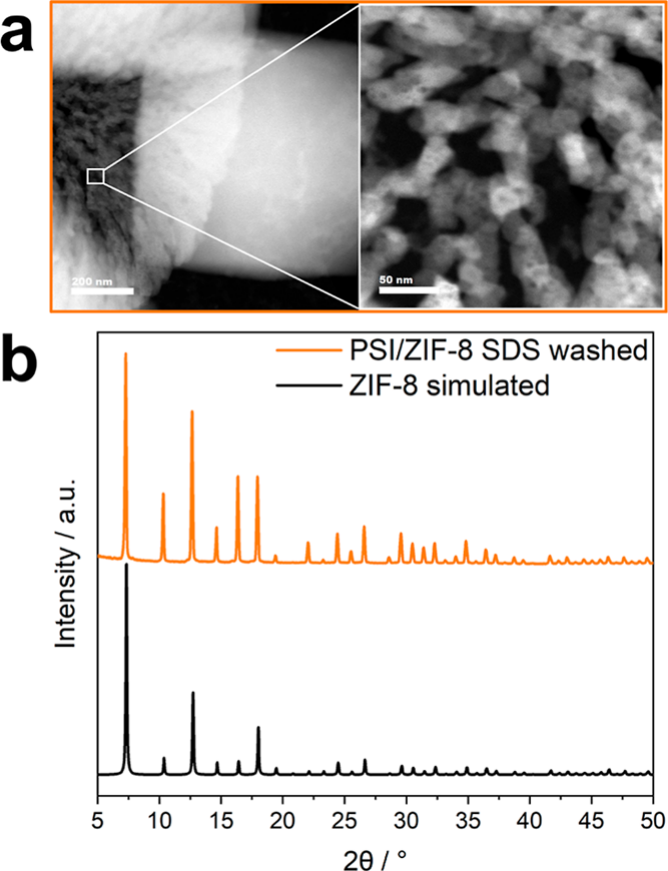
(a) HAADF-STEM images of as-synthesized PSI/ZIF-8, with the inset (right image) showing PSI units immobilized on the surface of ZIF-8 crystals. PSI is heterogeneously distributed and forms aggregates, (b) PXRD of PSI/ZIF-8 after SDS washing and a simulated ZIF-8 pattern as reference.

**Figure 5:**
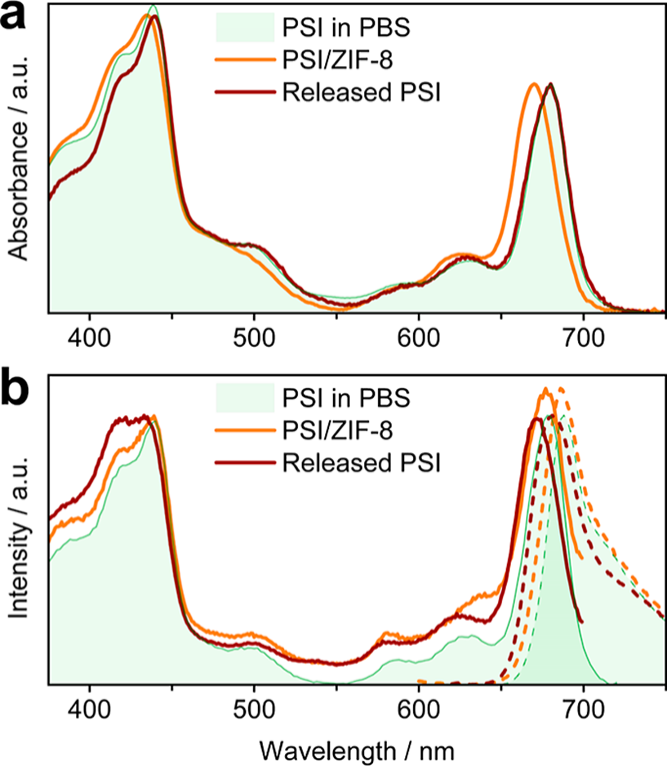
(a) Normalized (DR)-UV/Vis spectra of PSI in phosphate buffer (green shaded area), encapsulated in ZIF-8 (orange line) and after release from the ZIF-8 support (brown line). (b) Normalized fluorescence (dashed lines) and fluorescence excitation (full lines) of PSI in phosphate buffer (green shaded area, λ_Ex_ = 425 nm, λ_Em_ = 730 nm), encapsulated in ZIF-8 (orange, λ_Ex_ = 440 nm, λ_Em_ = 740 nm) and after release from the ZIF-8 support (brown, λ_Ex_ = 430 nm, λ_Em_ = 720 nm).

Digestion of PSI/ZIF-8 in acetate buffer (500 mM, pH 5.3) resulted in near-quantitative recovery of the solvated PSI UV/Vis spectrum (Figure 5a), with the B- and Q-band spectral shapes, positions and intensities almost perfectly mirroring those of pristine PSI apart from a minor loss in Q-band intensity (∼3%). However, as with PSI@ZIF-8, fluorescence and excitation spectra demonstrate a blueshift of the Q-band and line-broadening (Figure 5b). Although this effect is less drastic than for the encapsulated sample (Q-band shift of PSI released from PSI@ZIF-8: −12 nm (em.) and −8 nm (ex.), PSI/ZIF-8: −7 nm (em.) −6 nm (ex.)), these spectral alterations indicate that the buffer acidity during ZIF-8 digestion is sufficient to effect a small amount of pigment and Mg leaching, presumably from the surface exposed chlorophylls. Details of all spectral data (UV/Vis and fluorescence excitation and emission) are documented in Table S2.

Overall, the interfacing procedure to form PSI/ZIF-8 perturbed the PSI units considerably less than the mineralization process. Although the UV/Vis spectra shifted towards higher energies upon ZIF-8 interfacing, potentially due to strain, dielectric changes induced by the matrix and strong PSI/ZIF-8 interactions, PSI could be recovered globally unharmed. The observed changes to the fluorescence spectra reveal that a small fraction of the PSI chlorophylls remain vulnerable to pH induced decomposition. Still, this contrasts with the encapsulated PSI, where the fluorescence and excitation spectra were more significantly altered.

In summary, the presented data demonstrate that PSI encapsulated within ZIF-8 is significantly more stable against external media. For PSI@ZIF-8, only surface-bound PSI units were removed by SDS washing, while the encapsulated units remained unaffected due to the MOF shielding. On the other hand, PSI/ZIF-8 is entirely surface-bound and can readily be removed with an SDS wash. While PSI@ZIF-8 is clearly more chemically robust, the fluorescence data reveal a larger degree of chlorophyll decomposition upon mineralization as opposed to the milder surface interfacing procedure. Nevertheless, considering the UV/Vis spectra, intact PSI remains the majority species for both PSI@ZIF-8 and PSI/ZIF-8. PSI’s large and complex structure (∼600 kDa), composed of multiple protein subunits, pigments, and cofactors, makes it prone to structural perturbations during encapsulation.^58^ Such challenges have been documented before; for instance, cytochrome *c* encapsulated in ZIF-8 showed significant changes in secondary structure due to interactions with MOF precursors.^59^ Similarly, catalase adsorbed on ZIF-8 exhibited fluorescence shifts indicating perturbation of its tertiary structure.^60^ While such effects or reduced accessibility to cofactors and substrates might compromise the photochemical activity of PSI, the protective effects of ZIF-8 as a scaffold or support could enhance its stability against environmental stressors and challenging conditions.^58^ In previous studies, enzymes such as horseradish peroxidase (∼44 kDa) and glucose oxidase (∼160 kDa), encapsulated in ZIF-8 exhibited enhanced thermal and chemical stability due to the protective environment provided by the MOF.^61,62^ For the large protein catalase (∼240 kDa), ZIF-8 encapsulation improved thermal stability but required specific synthesis conditions to avoid structural damage.^63^ These findings underscore the need to balance stability with structural preservation when designing such composites. Other frameworks, such as those from the MIL family (e.g., MIL-101, MIL-100) or pyrene- and porphyrin-based MOFs (e.g., NU-1000, PCNs), could also be investigated to further elucidate how scaffold or support properties influence the structural impact on PSI.

### PSI Media Tolerance

To understand the origin of the discrepancies between entrapped and immobilized PSI and elucidate the impact of the mineralization process, we investigated the effects of the MOF building blocks, washing and digestion media on PSI integrity. For this purpose, changes to the PSI in different chemical environments were monitored via UV/Vis spectroscopy for ∼300 min in aqueous-based media containing Zn^2+^, 2-MeIm, acetate buffer or SDS (details in the experimental section, Figure S10). PSI absorption in 50 mM PBS at pH 7.4 was used as a reference since the buffer is widely used for storing proteins due to its ability to maintain a stable pH environment, making it a reliable baseline for comparative analyses.^22,64^ Anticipated scenarios include peak shifting or broadening, which may result from coordinating agents affecting the UV/Vis activity of PSI,^65,66^ while decreases in absorption intensity often stem from PSI precipitation or Mg leaching.^67–69^

First, the effect of the presence of ZIF-8 building blocks, at concentrations similar to those used for encapsulation, was monitored. Upon Zn^2+^ addition (0.27 M Zn­(OAc)_2_ solution), no qualitative changes in the features of the spectra were observed (Figure S10a). Nonetheless, PSI has a low isoelectric point, potentially favoring interactions with soft and hard Lewis acids such as metal cations.^70,71^ Thus, we performed ζ-potential measurements of PSI in the presence of Zn^2+^ (Figure S11). A ζ-potential of −22.7 ± 2 mV at pH 7 was recorded for pristine PSI, which is in good agreement with literature values.^72^ Adding a Zn^2+^ solution resulted in an increase to −17.2 ± 1 mV, indicating effective electrostatic interactions, which facilitate the encapsulation and immobilization of PSI.

Addition of the linker 2-MeIm (1.49 M, pH 11) to the PSI solution induced a modest hypsochromic shift of 2 nm but no significant changes to the rest of the spectrum (Figure S10b). Fluorescence emission and excitation spectra of PSI in the presence of 2-MeIm similarly revealed ∼4 nm hypsochromic spectral shifts (Figure S12), as well as noticeable alterations to the B-band vibronic structure.^67^ This is consistent with the fluorescence spectra of the digested PSI/ZIF-8 material and suggests potential interactions between the PSI enzyme and the linker (Figure 5b). The interaction mechanism is tentatively ascribed to a 2-MeIm-PSI coordination of peripheral chlorophyll units via Mg^2+^-N bonding, as observed in prior work on Mg porphyrins.^73^ Recent molecular dynamics simulations also suggested a coordination between methyl imidazolate and Mg^2+^ of PSI.^42^ Nonetheless, the modest alteration of the spectrum hints at a mono-coordination, as steric hindrance from the methyl group and the protein scaffold may limit further reactivity.^54,73–76^

Next, PSI stability was investigated in 500 mM acetate buffer (pH 5.3), as an effective medium for ZIF-8 digestion (Figure S10c). Although enzymes often unfold or denature in acidic conditions,^54,77^ the PSI spectra remain relatively unaltered beyond a small reduction in the overall absorption strength over time. This is in accordance with the general sensitivity of enzymes towards lower pH values, as they can induce structural changes, e.g., through hydrogen bonds.^14,54^ These minor pH-induced protein alterations might induce aggregation, precipitation or dissociation of a minor Chl_a_ population from the PSI structure, followed by Mg leaching and pheophytin formation.^54,78^

Strong interactions between PSI and the detergent SDS were found, as its presence induced dramatic alterations in the band features. Initially (<30 min), the PSI spectrum remains unchanged, displaying the characteristic Chl_a_ features. However, over time, SDS induces significant spectral alterations resembling those observed during MOF encapsulation and digestion—likely linked to the formation of decomposition products such as pheophytin (Figure S10d). This suggests that PSI structure is not retained in the presence of SDS. The detergent properties of SDS likely disrupt the protein-pigment interactions, further supporting the protective role of ZIF-8 in preserving PSI integrity, as no major spectral changes were recorded post-washing for the encapsulated hybrid (Figure S5).

In summary, PSI exhibits good overall tolerance to ZIF-8 building blocks and the digestion medium. The maintenance of spectral features aligns with the principle that simple additives typically do not largely disrupt the structural integrity of biomacromolecules. Several studies on membrane proteins have shown that they can often tolerate the presence of salts or small organic molecules without significant loss of function.^79,80^ PSI’s stability in acetate buffer further aligns with its broad pH tolerance, which, while superior to many other enzymes, is common in membrane proteins adapted to diverse cellular environments.^81–83^ Likewise, PSI’s strong interaction with the detergent SDS is a phenomenon observed for many (membrane) proteins, which often completely denature in SDS.^84–86^

These results support our findings that the increased damage observed in entrapped PSI, compared to the immobilization process, likely arises from steric and mechanical constraints as well as different dielectric strength environment applied within the MOF network. Moreover, the hypothesis that the decomposition of a small fraction of PSI chlorophylls observed for both PSI@ZIF-8 and PSI/ZIF-8 can be attributed to the acetate digestion buffer was affirmed.

## Conclusions

Our study aimed to enhance our understanding of the synthetic mechanisms, the driving forces behind hybridization processes, and the influence of ZIF-8 on PSI towards creating a bio-hybrid material that leverages the unique properties of the enzymes and the protective, structural benefit of the MOF. We established a facile one-pot scaffolding process to incorporate PSI into ZIF-8. Our investigations revealed that release of the enzyme from the MOF scaffold results in global PSI preservation yet accompanied by partial pheophytin formation likely originating from the encapsulation. We relate this to stress and strain on the protein, potentially caused by MOF confinement. In particular, the linker 2-MeIm was identified as having an impact on the PSI, presumably by coordinating with the Mg^2+^ ions of peripheral chlorophylls. In parallel, a MOF-surface impregnation approach was found to have less impact on the enzyme, as indicated by fluorescence spectroscopy. However, the enzyme is easily washed off the support and the distribution is inhomogeneous. HAADF-STEM images confirmed the formation of the PSI@ZIF-8 composite, marking the first imaging of PSI inside a MOF. These findings on PSI-based scaffolded bio-nanomaterials pave the way to further understanding and future exploitation of the high-performance, light-harvesting enzyme for practical applications.

## Supplementary Material



## Notes

Additional data include supplementary figures and tables (PDF).
