# Supplementary material for: Photosystem I and ZIF‑8 Interfacing: Entrapment and Immobilization
Source: Inorg Chem. 2025 May 20;64(21):10369–78. doi: 10.1021/acs.inorgchem.4c05441 (PMC12135045; doi:10.1021/acs.inorgchem.4c05441)
Supplement: Supplementary file 1 [file ic4c05441_si_001.pdf]

Supporting Information

Photosystem I and ZIF-8 interfacing: entrapment and immobilization

Kathrin L. Kollmannsberger,[a] Sarah V. Dummert,[a] Erling Thyrhaug,[b] Pritam Banerjee,[c] Feng Liu,[d] Dario Leister,[d] Joerg Jinschek,[c] Jürgen Hauer*,[b] Roland A. Fischer*,[a] and Julien Warnan*[a]

a Chair of Inorganic and Metal-Organic Chemistry, Department of Chemistry, TUM School of Natural Sciences, Technical University of Munich, Lichtenbergstr. 4, 85748, Garching, Germany.

b Professorship of Dynamic Spectroscopy, Department of Chemistry and Catalysis Research Center (CRC), TUM School of Natural Sciences, Technical University of Munich, Lichtenbergstr. 4, 85748 Garching, Germany.

c National Centre for Nano Fabrication and Characterization (DTU Nanolab), Technical University of Denmark, Fysikvej 307, DK-2800 Kongens Lyngby, Denmark.

d Faculty of Biology, Ludwig-Maximilians-Universität München, Großhaderner Str. 2-4, 82152 Planegg-Martinsried, Germany.

* Corresponding Authors: juergen.hauer@tum.de, roland.fischer@tum.de, julien.warnan@tum.de

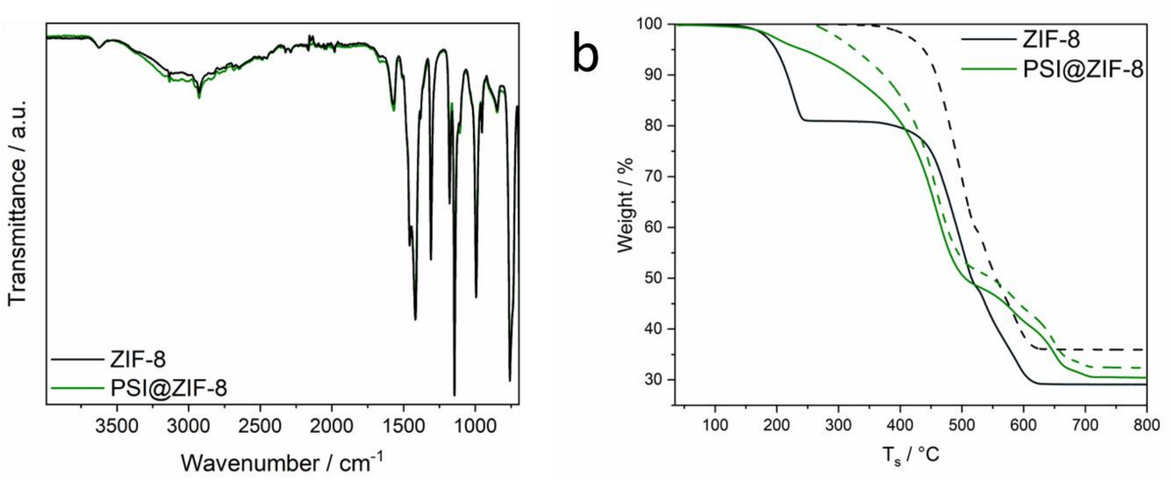

Figure S1: **a** FTIR spectra and **b** TGA curves of pristine ZIF-8 (black) and PSI@ZIF-8 (green). In **b** the dashed lines represent the TGA profiles normalized to the respective mass at 260 °C, excluding the weight loss from volatile residues.

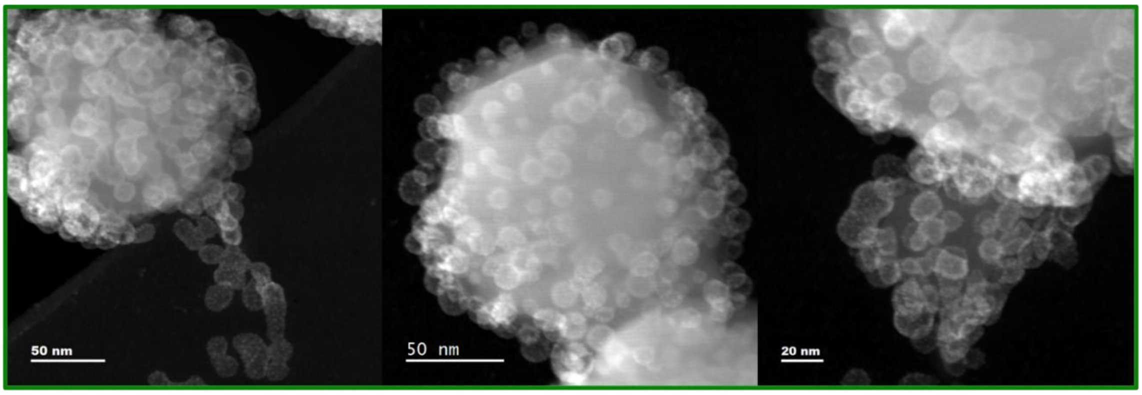

Figure S2: HAADF-STEM images of PSI@ZIF-8 with PSI visibly decorated on the surface of ZIF-8 crystals.

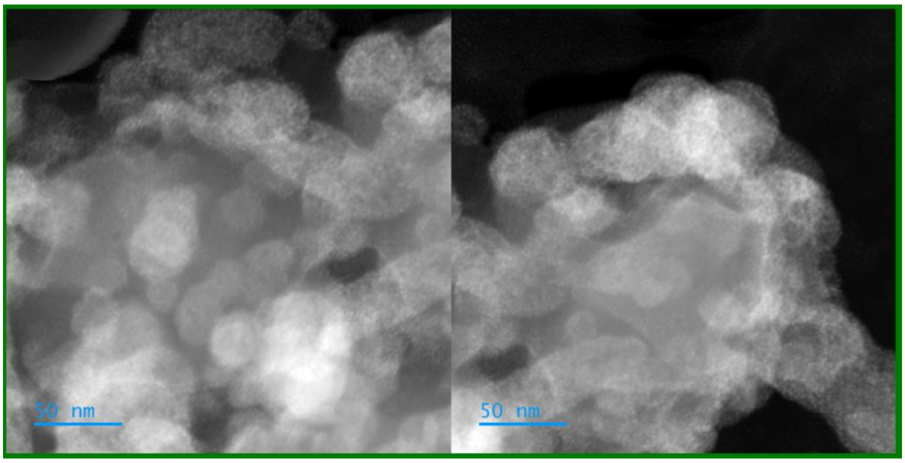

Figure S3: HAADF-STEM images of PSI@ZIF-8 with PSI visibly encapsulated in ZIF-8 crystals. Encapsulated PSI appears as bright regions within ZIF-8.

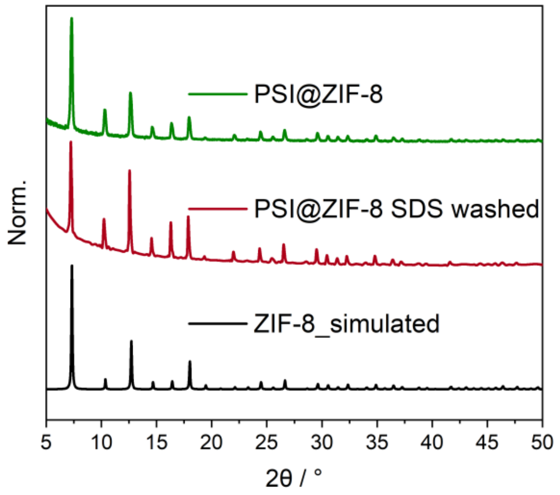

Figure S4: PXRD of PSI@ZIF-8 after synthesis (green, top trace), and after SDS washing (red, middle trace), and of the simulated ZIF-8 pattern (black, bottom trace).

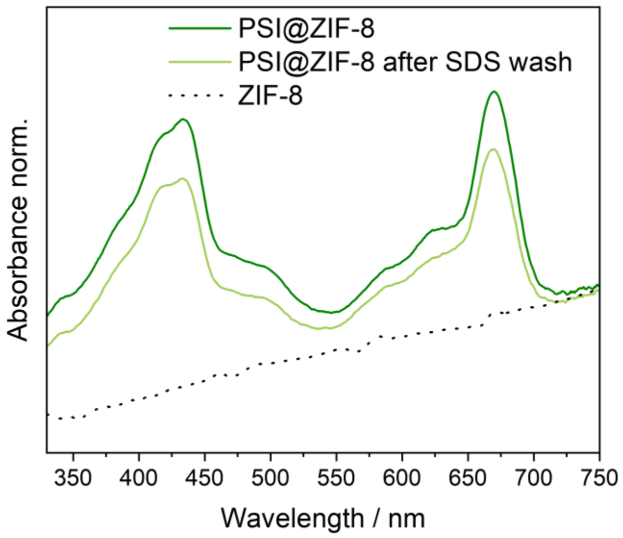

Figure S5: DR-UV/Vis spectra of PSI@ZIF-8 before (dark green) and after (light green) SDS washing. The DR-UV/Vis spectrum of pristine ZIF-8 (black, dotted) is shown for comparison.

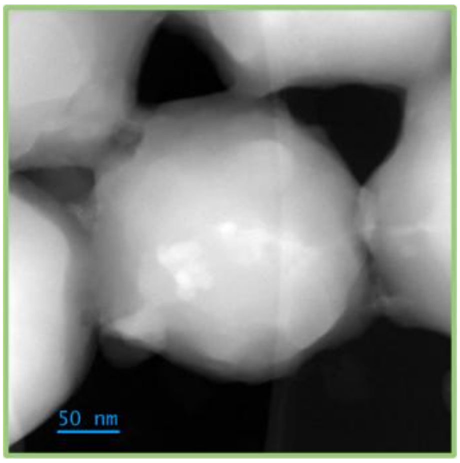

Figure S6: HAADF-STEM image of PSI@ZIF-8 after SDS washing.

Table S1: Wavelengths [nm] and normalized Intensities [a.u.] of B- and Q-bands in UV/Vis, fluorescence excitation and fluorescence emission spectra of pristine PSI in PBS, PSI@ZIF-8 and PSI released from PSI@ZIF-8. For all bands, the shifts in wavelengths of maximum signal intensity as well as the relative changes in signal intensity compared to the spectrum of pristine PSI are provided. The ratio of B- and Q-band intensities is given as well.

		**PSI in PBS**	**PSI@ZIF-8**	**Released PSI**
**UV/Vis**				
B band	Wavelength [nm]	438	433	438
	Absorbance, norm.	0.76	0.59	0.72
	WL shift [nm]	*origin*	-5	0
	Abs. Intensity [%]	*origin, ≙ 100%*	77	95
Q band	Wavelength [nm]	679	670	679
	Absorbance, norm.	0.56	0.56	0.54
	WL shift [nm]	*origin*	-9	0
	Abs. Intensity [%]	*origin, ≙ 100%*	99	96
B:Q ratio	Abs. intensity ratio	1.35	1.06	1.33
**Excitation**				
B band	Wavelength [nm]	440	418	418
	Intensity, norm.	0.98	0.98	1
	WL shift [nm]	*origin*	-22	-22
	Intensity [%]	*origin, ≙ 100%*	100	102
Q band	Wavelength [nm]	678	668	670
	Intensity, norm.	1	1.07	0.61
	WL shift [nm]	*origin*	-10	-8
	Intensity [%]	*origin, ≙ 100%*	107	61
B:Q ratio	Intensity ratio	0.98	0.91	1.64
**Fluorescence**				
Q region	Wavelength [nm]	688	680	676
	WL shift [nm]	*origin*	-8	-12

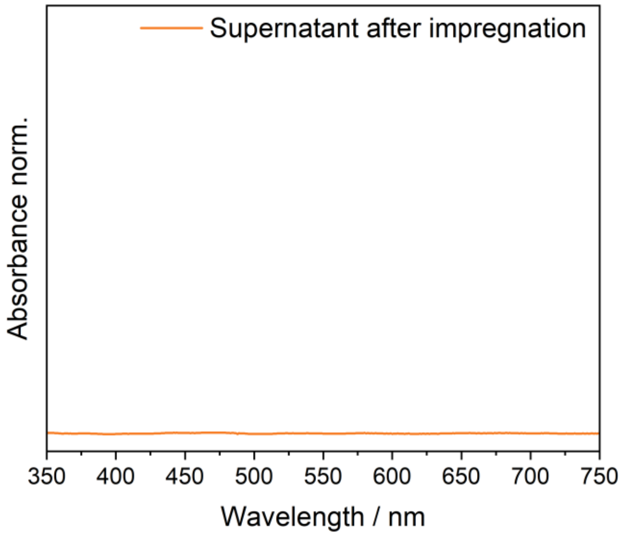

Figure S7: UV/Vis spectrum of the ZIF-8-PSI-impregnation supernatant.

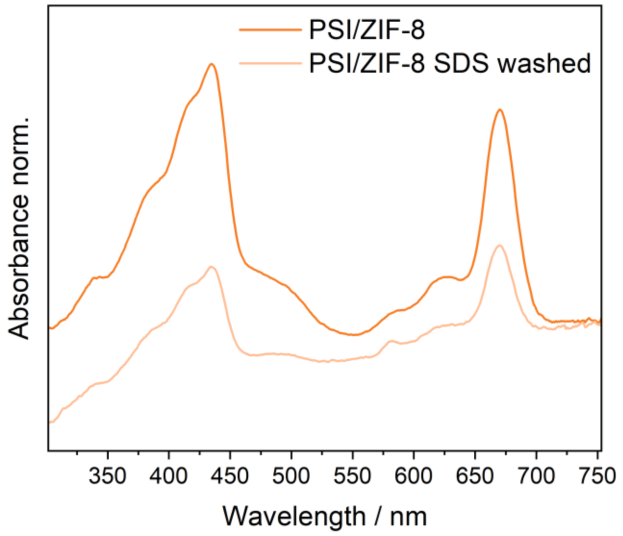

Figure S8: DR-UV/Vis spectra of PSI/ZIF-8 before (dark orange) and after (light orange) SDS washing.

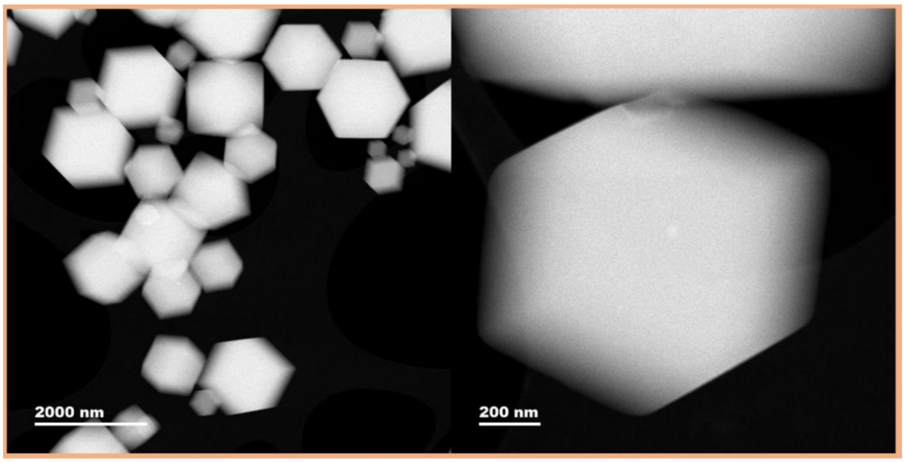

Figure S9: HAADF-STEM images of PSI/ZIF-8 after SDS washing.

Table S2: Wavelengths [nm] and normalized Intensities [a.u.] of B- and Q-bands in UV/Vis, fluorescence excitation and fluorescence emission spectra of pristine PSI in PBS, PSI/ZIF-8 and PSI released from PSI/ZIF-8. For all bands, the shifts in wavelengths of maximum signal intensity as well as the relative changes in signal intensity compared to the spectrum of pristine PSI are provided. The ratio of B- and Q-band intensities is given as well.

		**PSI in PBS**	**PSI/ZIF-8**	**Released PSI**
**UV/Vis**				
B band	Wavelength [nm]	438	435	440
	Absorbance, norm.	1.34	1.30	1.30
	WL shift [nm]	*origin*	-3	+2
	Abs. Intensity [%]	*origin, ≙ 100%*	97	97
Q band	Wavelength [nm]	680	670	680
	Absorbance, norm.	1	1	1
	WL shift [nm]	*origin*	-10	0
	Abs. Intensity [%]	*origin, ≙ 100%*	100	100
B:Q ratio	Abs. intensity ratio	1.34	1.30	1.30
**Excitation**				
B band	Wavelength [nm]	440	439	433
	Intensity, norm.	0.98	1	1
	WL shift [nm]	*origin*	-1	-7
	Intensity [%]	*origin, ≙ 100%*	102	102
Q band	Wavelength [nm]	678	677	673
	Intensity, norm.	1	1.1	0.99
	WL shift [nm]	*origin*	-1	-6
	Intensity [%]	*origin, ≙ 100%*	110	99
B:Q ratio	Intensity ratio	0.98	0.91	1.01
**Emission**				
Q region	Wavelength [nm]	688	686	681
	WL shift [nm]	*origin*	-2	-7

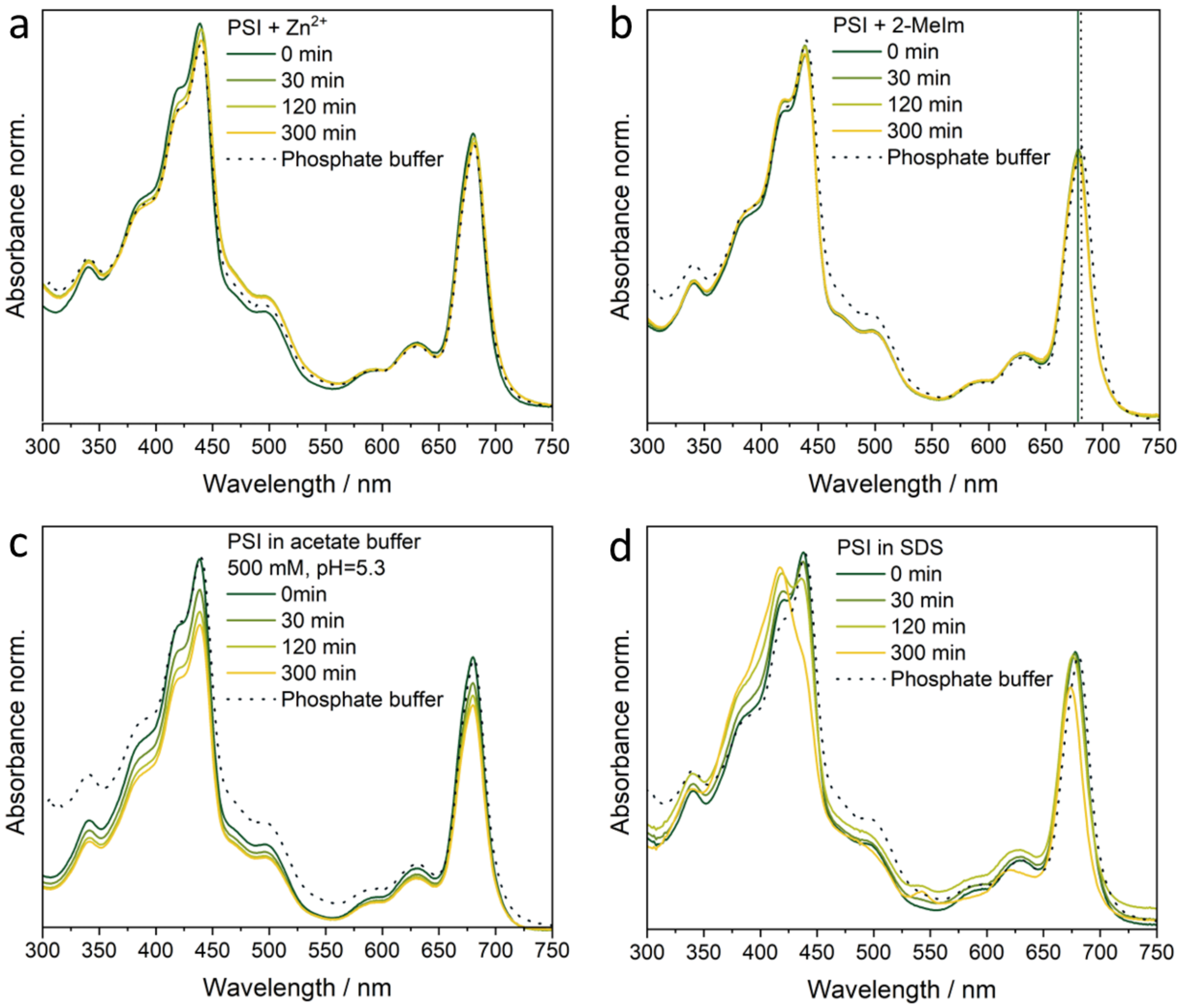

Figure S10: UV/Vis spectra of PSI **a** with Zn^{2+} (aq. 0.27 M $Zn(OAc)_2$), **b** with 2-MeIm (aq. 1.49 M), **c** in 500 mM acetate buffer (pH 5.3) and **d** in 4 wt% aq. SDS solution.

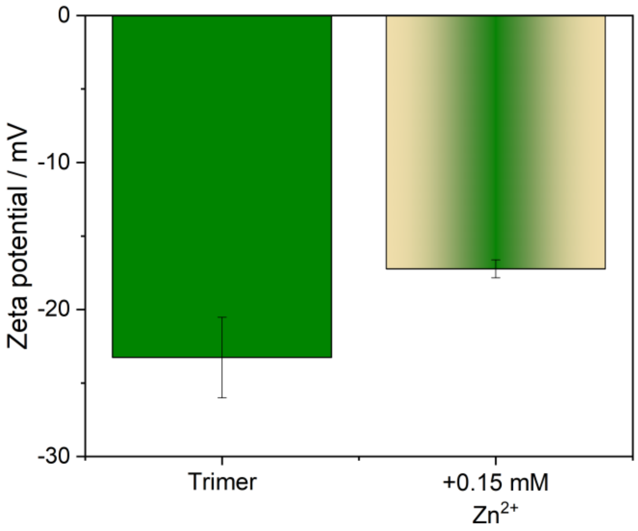

Figure 11: ζ-potential of PSI (denoted as Trimer) and PSI in the presence of Zn^{2+}(aq. 15 mM $Zn(OAc)_2$).

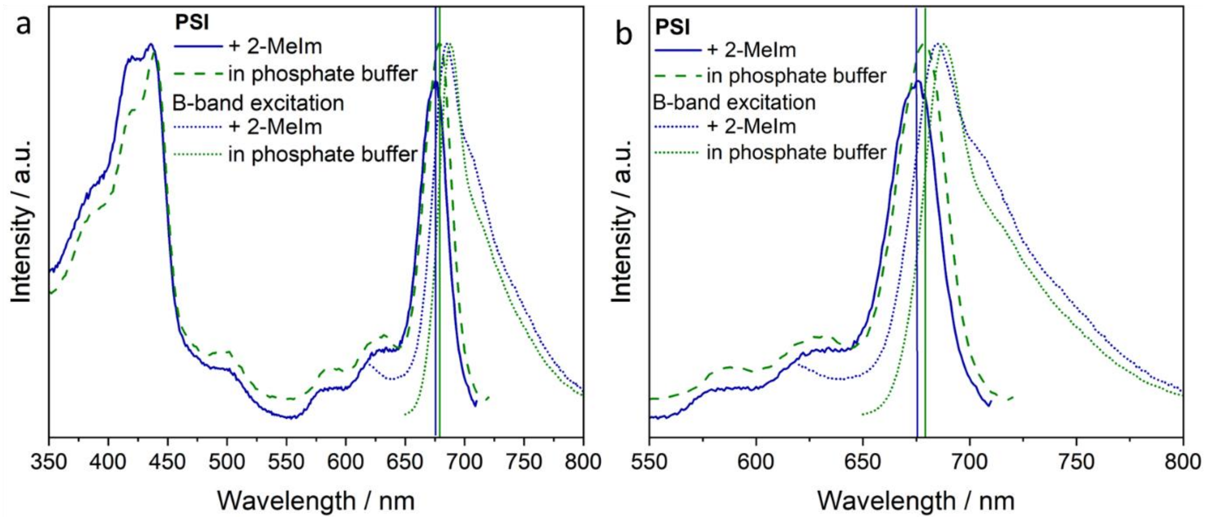

Figure S12: Excitation (detected @730 nm) and fluorescence (B-band excitation) spectra of PSI in 1.49 M 2-MeIm solution and in phosphate buffer (**a** whole spectra, **b** 550-800 nm inset). The vertical lines mark the maxima of the respective samples.
